# PD-L1 overexpression in EBV-positive gastric cancer is caused by unique genomic or epigenomic mechanisms

**DOI:** 10.1038/s41598-021-81667-w

**Published:** 2021-01-21

**Authors:** Hiroshi Nakano, Motonobu Saito, Shotaro Nakajima, Katsuharu Saito, Yuko Nakayama, Koji Kase, Leo Yamada, Yasuyuki Kanke, Hiroyuki Hanayama, Hisashi Onozawa, Hirokazu Okayama, Shotaro Fujita, Wataru Sakamoto, Zenichiro Saze, Tomoyuki Momma, Kosaku Mimura, Shinji Ohki, Akiteru Goto, Koji Kono

**Affiliations:** 1 Department of Gastrointestinal Tract Surgery, Fukushima Medical University School of Medicine, 1 Hikarigaoka, Fukushima, 960-1295 Japan; 2 Department of Medical Electrophysiology, Fukushima Medical University School of Medicine, Fukushima, 960-1295 Japan; 3 First Department of Surgery, Faculty of Medicine, University of Yamanashi, Yamanashi, 409-3898 Japan; 4 Department of Blood Transfusion and Transplantation Immunology, Fukushima Medical University School of Medicine, Fukushima, 960-1295 Japan; 5 Department of Cellular and Organ Pathology, Graduate School of Medicine, Akita University, Akita, 010-8543 Japan

**Keywords:** Gastric cancer, Cancer microenvironment

## Abstract

Epstein-Barr virus-positive gastric cancer [EBV (+) GC] is a distinct GC subtype with unique genetic and epigenetic aberrations. Here, we examined resected GC samples and publicly available microarray data and The Cancer Genome Atlas (TCGA) database to identify the mechanism underlying overexpression of PD-L1 in EBV (+) GC. We found that high levels of PD-L1 overexpression in EBV (+) GC were caused by focal amplification of *CD274*. By contrast, relatively high expression of PD-L1 in tumor tissue and infiltrating immune cells correlated with CD8 lymphocyte infiltration and IFN-γ expression via IRF3 activation. Since we reported previously that PD-L1 expression is associated both with the presence of CD8 T cells in the tumor microenvironment and with IFN-γ expression in GC, we examined a database to see whether IFN-γ-associated overexpression of PD-L1 plays a significant role in EBV (+) GC. Immunohistochemical staining showed that expression of the IRF3 signature in clinical GC samples was higher in EBV (+) than in EBV (−) cases. The data presented herein reveal a unique dual mechanism underlying PD-L1 overexpression in EBV (+) GC: high focal amplification of *CD274* or IFN-γ-mediated signaling via activation of IRF3.

## Introduction

Epstein-Barr virus (EBV)-positive (+) gastric cancer (GC), a distinct GC subtype defined by EBV infection, accounts for nearly 10% of GC^1,2^. EBV (+) GC develops due to accumulation of both genetic and epigenetic modifications^2^. A striking epigenetic feature of EBV (+) GC is promotor CpG hypermethylation^3^. Another epigenetic feature is EBV-encoded microRNAs; indeed, we recently reported that EBV-encoded microRNAs play important regulatory roles in EBV-mediated gastric carcinogenesis^4^. The common genetic features of EBV (+) GC were reported in large-scale genome sequencing studies, including The Cancer Genome Atlas (TCGA)^1,3,5^. Unique characteristics of EBV (+) GC include frequent mutations in phosphatidylinositol-4,5-bisphosphate 3-kinase catalytic subunit alpha (*PIK3CA*) and the AT-rich interactive domain 1A (*ARID1A*), lack of *TP53* mutations, and amplification of Janus Activating Kinase 2 (*JAK2*) and *CD274*/*PDCD1LG2*, leading to overexpression of programmed death ligand-1 (PD-L1)/PD-L2.

Immune checkpoint monoclonal antibodies (mAbs) targeting the PD-1 axis have a positive effect in heavily pretreated GC patients^6^. Evaluation of the therapeutic effects of anti-PD-1 mAbs against GC shows that patients with EBV (+) metastatic GC, as well as a high PD-L1 combined positive score (CPS) and tumors showing high microsatellite instability (MSI), have a favorable response^7^. Since there is a strong correlation between EBV (+) and PD-L1 positivity, patients with EBV (+) GC benefit from anti-PD-1 treatment; indeed, EBV (+) and PD-L1 positivity predict better outcomes. Regarding PD-L1/CD274, we previously reported that focal and high level amplification of *CD274* results in high PD-L1 expression in a small subset of small cell lung cancers (SCLC)^8^. By contrast, non-focal, arm-level copy number gain of 9p did not lead to extremely high PD-L1 expression in SCLC, suggesting that the mechanism underlying PD-L1 overexpression in EBV (+) GC requires further copy number amplification, including that of the *CD274* locus. Additionally, we reported that PD-L1 expression by GC cells correlates significantly with the presence of CD8 T cells in the tumor microenvironment and with interferon-γ (IFN-γ) expression^9,10^. IFN-γ-mediated upregulation of *CD274* was also observed in EBV-associated B cell lymphoma, where it inhibited killing of infected cells by cytotoxic T cells expressing PD-1 ligand^11^. These results suggest the possibility that PD-L1 overexpression associated with the presence of CD8 T cells and IFN-γ occurs preferentially in EBV (+) GC due to virus-related immune evasion. It appears that IFN-γ-mediated overexpression of PD-L1 occurs via a mechanism different from PD-L1 overexpression mainly due to amplification of *CD274*. Therefore, the mechanism underlying regulation of PD-L1 expression in EBV (+) GC should be considered separately.

EBV infection triggers innate antiviral immune responses in infected cells, which produce pro-inflammatory cytokines and type I IFNs^12^. During this response, interferon regulatory factor 3 (IRF3) plays a pivotal role. IRF3 is a transcription factor that is usually activated in tumor cells through phosphorylation, dimerization, or nuclear translocation. IRF3 phosphorylation (pIRF3) in EBV-infected cells is triggered by EBV-encoded small RNAs (EBERs; e.g., EBER1 and EBER2) through the toll-like receptor (TLR) or RIG-I signaling pathways^12,13^. IRF3 also plays a role in adaptive T cell immune responses, as well as in innate immune responses^14^. A previous study demonstrates that mice deficient in IRF3 show impaired expression of IFN-γ by memory T cells during T cell responses to virus infection, suggesting that IRF3 is a positive regulator of oncogenic pathways involving IFN-γ^15^. Indeed, pIRF3 induces production of IFN-γ from T cells, and PD-L1/CD274 expression correlates significantly with IRF3 activation in malignant melanoma^16,17^. These findings provide a model that explains overexpression of PD-L1 in EBV (+) GC: IRF3 is activated in EBV (+) GC, resulting overexpression of PD-L1 via IFN-γ.

Here, we investigated the unique mechanisms underlying PD-L1 overexpression in EBV (+) GC through data analysis using different approaches^18^. First, we found that PD-L1 overexpression was observed in EBV (+) GC, suggesting due to *CD274* copy number aberrations in clinical samples. We then confirmed this using publically available data. Second, we assessed the hypothesis that IRF3 is activated by EBV infection, thereby driving PD-L1 overexpression in EBV (+) GC via IFN-γ. Activation of IRF3 was investigated using public databases and clinical samples.

## Results

### Histological examination of PD-L1 upregulation in EBV (+) GC

**Figure 1:**
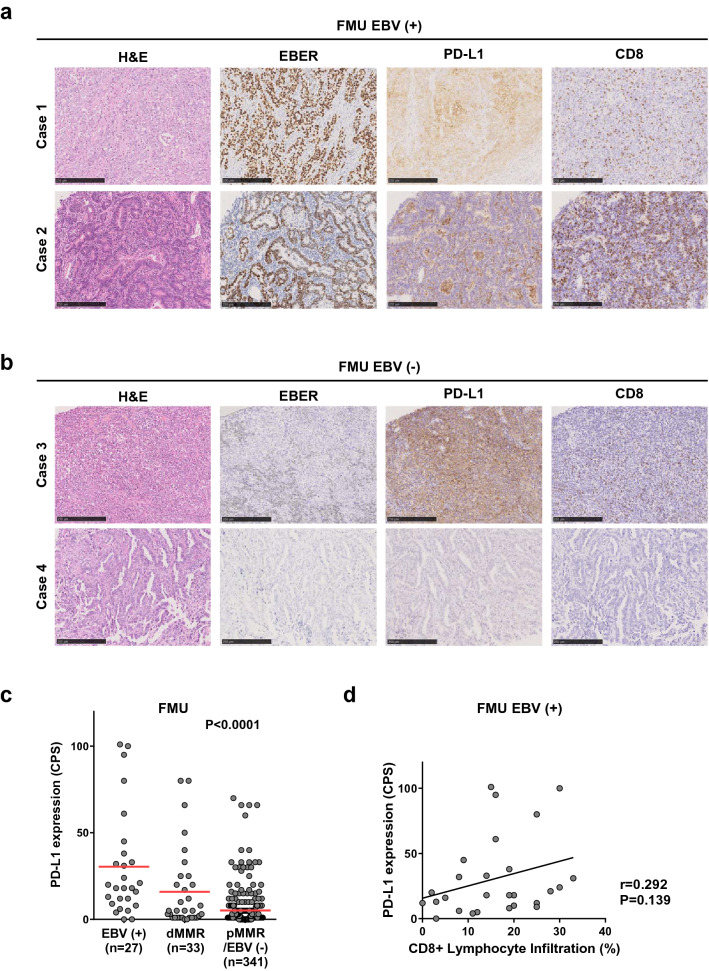

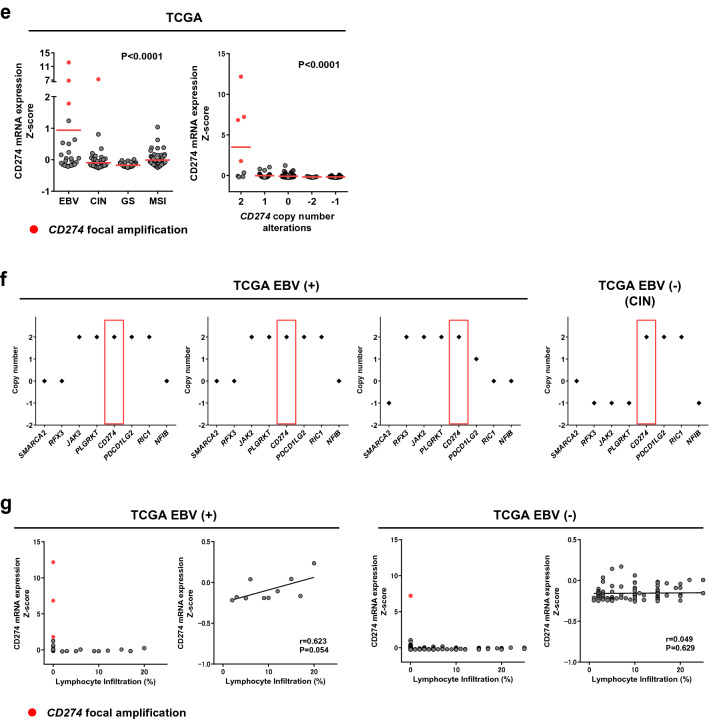
Histological comparison of PD-L1/CD274 expression, copy number aberrations, and lymphocyte infiltration in EBV (+) gastric cancer (GC). **(a)** Representative images showing IHC staining for HE, EBER-ISH, PD-L1, and CD8 T cells in EBV (+) GC from the Fukushima Medical University (FMU) cohort. Case 1 with high PD-L1 (CPS = 95) and low percentage of infiltrating CD8 + lymphocytes (16%) and Case 2 with high PD-L1 (CPS = 100) and high percentage of infiltrating CD8 + lymphocytes (30%) expressions are shown. Scale bars = 250 μm. HE, Hematoxylin/Eosin; EBER-ISH, EBV-encoded small RNA in situ hybridization. **(b)** Representative images showing IHC staining for HE, EBER-ISH, PD-L1, and CD8 T cells in EBV (−) GC from the FMU cohort. Case 3 with relatively high PD-L1 (CPS = 66) and low percentage of infiltrating CD8 + lymphocytes (10%) and Case 4 with low PD-L1 (CPS = 0) and low percentage of infiltrating CD8 + lymphocytes (0%) expressions are shown. Scale bars = 250 μm. **(c)** PD-L1 expression among EBV (+), dMMR, and pMMR/EBV (−) GC cases in the FMU cohort. **(d)** Correlation between CD8 + lymphocyte infiltration and PD-L1 expression in FMU EBV (+) GC cases (n = 27). **(e)** Differential expression of *CD274* mRNA among EBV (+), CIN, GS, and MSI GC in TCGA (left), and comparison of *CD274* mRNA expression and copy number alterations in GC cases from TCGA. Three cases of EBV (+) GC and one case of CIN showed focal and high level *CD274* amplification, resulting in high *CD274* mRNA expression (indicated by the red dot). **(f)** Copy number status of representative cancer-related genes that mapped telomeric and centromeric to *CD274* on 9p24.1. Four cases from TCGA (indicated by red dot in **e**) exhibited focal and high level amplification of the segment containing *CD274*. **(g)** Comparison of *CD274* mRNA expression and lymphocyte infiltration in EBV (+) (n = 24) and EBV (−) (n = 239) GC cases from TCGA. The red dot indicates a tumor with *CD274* focal amplification. Correlation between lymphocyte infiltration and *CD274* mRNA expression cases without *CD274* focal amplification and lymphocyte infiltration in EBV (+) (n = 10) and EBV (−) (n = 90) from TCGA. Expression of *CD274* mRNA positively correlated (albeit marginally) with lymphocyte infiltration in EBV (+) GC, but not in EBV (−) GC.

To investigate the mechanism underlying PD-L1 overexpression in EBV (+) GC, we first performed IHC staining for PD-L1 in the Fukushima Medical University (FMU) cohort that included 401 GC tumors (Table 1). The FMU cohort included 27 (6.7%) cases of EBV (+) and 33 (8.2%) cases of deficient mismatch repair (dMMR) GC, confirmed by EBER-ISH and IHC staining for MMR proteins, respectively (Fig. 1a,b and Table 1)^19^. Histological evaluation identified 12 (44%) cases of lymphoepithelioma-like carcinoma, 13 (48%) cases of conventional-type, and two (7%) cases of Crohn’s disease-like carcinoma (Supplementary Fig. S1). This analysis confirmed the previous observation that tumors with EBV (+) or dMMR are mutually exclusively, and that PD-L1 is significantly overexpressed in EBV (+) GC compared with dMMR or pMMR/EBV (−) GC (Fig. 1c). Because 9p24.1 amplification is one of the specific characteristics of genetic aberrations in EBV (+) GC, cases scored highest PD-L1 CPS (> 90) were suggested to be caused by *CD274* copy number aberrations. To explore the mechanism underlying PD-L1 overexpression in EBV (+) GC cases lacking *CD274* amplification, we attempted to determine whether PD-L1 expression is associated with the presence of CD8 T cells. Because we previously reported that PD-L1 expression by GC cells correlates significantly with the presence of CD8 T cells in the tumor microenvironment and with expression of IFN-γ^9,10^, we first decided to investigate the correlation between PD-L1 expression and the presence of CD8 T cells using histological evaluation focusing on in EBV (+) GC (Fig. 1a and Supplementary Fig. S2). Analysis of EBV (+) GC (n = 27) revealed that PD-L1 expression in EBV (+) GC correlated positively with CD8 + lymphocyte infiltration, although the result did not reach statistical significance (probably due to the small number of samples in the FMU cohort) (Fig. 1d). These results suggest that PD-L1 overexpression with the presence of CD8 T cells and IFN-γ which observed in GC cells were also observed in EBV (+) GC and, importantly, cases expected to harboring *CD274* amplifications were independent of CD8 (+) lymphocyte infiltration.

**Table 1:** Clinicopathological characteristics of gastric cancer patients from FMU cohort.

Characteristics	Total (n = 401)	EBV (+) (n = 27)	dMMR (n = 33)	pMMR/EBV (−) (n = 341)
**Age-year**				
Mean	67.7	66.5	75.8	67.1
Range	30–92	48–81	59–92	30–91
**Gender-no. (%)**				
Male	283 (71)	20 (74)	21 (64)	242 (71)
Female	118 (29)	7 (26)	12 (36)	99 (29)
**Tumor location**				
Upper	128 (32)	17 (63)	3 (9)	108 (32)
Middle	136 (34)	7 (26)	8 (24)	121 (35)
Lower	124 (31)	1 (4)	21 (64)	102 (30)
Whole	6 (1)	1 (4)	0	5 (1)
N/A	7 (2)	1 (4)	1 83)	5 (1)
**Histological type-no. (%)**				
Differentiated	203 (51)	11 (41)	21 (64)	176 (52)
Undifferentiated	191 (49)	16 (59)	12 (36)	165 (48)
**Histological type-no. (%)**				
Conventional-type adenocarcinoma	–	13 (48)	–	–
Crohn's disease-like lymphoid reaction	–	2 (7)	–	–
Lymphoepithelioma-like carcinoma	–	12 (44)	–	–
**TNM stage-no. (%)**				
I	219 (55)	11 (41)	16 (48)	192 (56)
II	79 (20)	6 (22)	8 (24)	65 (19)
III	70 (17)	6 (22)	6 (18)	58 (17)
IV	33 (8)	4 (15)	4 (12)	26 (8)
**LN metastasis-no. (%)**				
Positive	247 (62)	11 (41)	12 (36)	132 (39)
Negative	154 (38)	16 (59)	21 (64)	209 (61)
**Lymphatic invasion-no. (%)**				
Present	218 (54)	14 (52)	20 (60)	184 (54)
Absent	182 (45)	13 (48)	12 (36)	157 (46)
N/A	1	0	1 (3)	0
**Venous invasion-no. (%)**				
Present	228 (57)	21 (78)	20 (60)	187 (55)
Absent	172 (42)	6 (22)	12 (36)	154 (45)
N/A	1 (1)	0	1 (3)	0
**Mismatch repair (MMR)-no. (%)**				
Deficient MMR	33 (8.2)	0	33 (100)	0
Proficient MMR	368 (92)	27 (100)	0	341 (100)
**Epstein-Barr virus (EBV)-no. (%)**				
Positive	27 (6.7)	27 (100)	0	0
Negative	374 (93.3)	0	33 (100)	341 (100)

To further confirm those findings, we analyzed a TCGA stomach adenocarcinoma tissue dataset (n = 269) and found that cases with *CD274* amplification showing high expression of *CD274* mRNA were common in EBV (+) GC (Fig. 1e). To examine *CD274* amplification status, we determined the copy number of representative cancer-related genes that mapped telomeric and centromeric to *CD274*. Three cases of EBV (+) GC and one case of chromosomal instability (CIN) GC with high *CD274* mRNA expression exhibited focal and high level amplification of the segment containing *CD274* (Fig. 1f). By contrast, cases with relatively high expression of *CD274* mRNA exhibited non-focal and arm-level copy number gain of 9p (Supplementary Fig. S3). Next, we obtained lymphocyte infiltration data from TCGA pathological reports and attempted to confirm the positive correlation between *CD274* expression and lymphocyte infiltration. Consistent with our cohort, cases with *CD274* focal amplification lacked lymphocyte infiltration in both EBV (+) and EBV (−) GC, and *CD274* expression was marginally associated with lymphocyte infiltration in EBV (+) GC cases without *CD274* focal amplification and lymphocyte infiltration (Fig. 1g). However, *CD274* expression was not associated with lymphocyte infiltration in EBV (−) GC cases without *CD274* focal amplification and lymphocyte infiltration (Fig. 1g). This pathological analysis is in line with our previous studies, and suggests that there are two mechanisms underlying PD-L1 overexpression in EBV (+) GC: one due to focal amplification of *CD274* and the other due to lymphocyte (CD8 T cells) infiltration^9,10^.

## Histological feature of PD-L1 upregulation in EBV (+) GC

To further explore the mechanisms underlying PD-L1 overexpression in EBV (+) GC, we focused on the role of IFN-γ produced by tumor-infiltrating lymphocytes, including CD8 T cells, during virus infection^9,12^. Based on our recent finding that the IFN-γ gene signature correlates significantly with expression of *CD274* mRNA in GC, we next attempted to investigate the effect of the IFN-γ signature on PD-L1/*CD274* expression in EBV (+) GC^10^.

**Figure 2:**
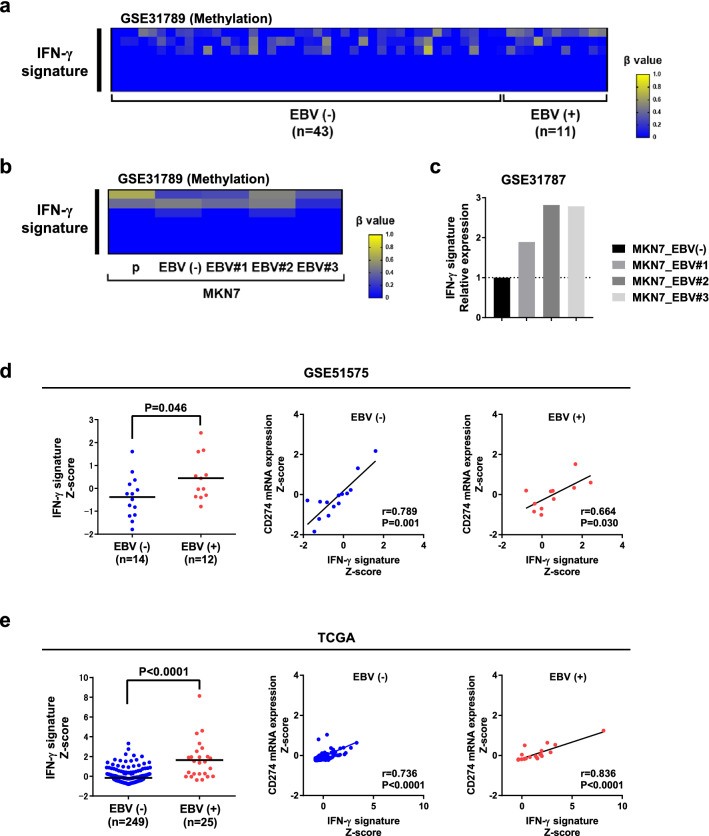
DNA methylation status of IFN-γ signature genes and correlation between IFN-γ signature gene expression and *CD274* mRNA expression in EBV (+) GC. **(a)** DNA methylation status of IFN-γ signature genes (six genes) in EBV (−) (n = 43) and EBV (+) (n = 11) GC (GSE31789). DNA hypermethylation was not observed in IFN-γ signature genes. The β value (0.00 to 1.00) reflects the methylation level at individual CpG sites. **(b)** DNA methylation status of IFN-γ signature genes (six genes) among parent (MKN7 p), mock [MKN7 EBV (−)], and EBV-infected clones (MKN7 EBV#1, EBV#2, and EBV#3) of MKN7 cells (GSE31789). DNA hypermethylation of IFN-γ signature genes was not observed. The β value (0.00 to 1.00) reflects the methylation level at individual CpG sites. **(c)** Comparison of IFN-γ signature gene expression between mock and EBV-infected MKN7 cell clones (GSE31787). The IFN-γ signature was upregulated in EBV-infected MKN7 cells compared with control cells. **(d)** Differential expression of IFN-γ signature genes between EBV (−) and EBV (+) GC (left, GSE51575). IFN-γ signature genes were upregulated in EBV (+) GC compared with EBV (−) GC (P = 0.046). Comparison of IFN-γ signature and *CD274* mRNA expression in EBV (+) and EBV (−) GC (right two panels). Expression of *CD274* mRNA correlated positively with IFN-γ signature expression in EBV (+) GC (P = 0.030). **(e)** Differential expression of IFN-γ signature genes between EBV (−) and EBV (+) GC (left, TCGA). IFN-γ signature genes were upregulated in EBV (+) GC compared with EBV (−) GC (P < 0.0001). Comparison of IFN-γ signature and *CD274* mRNA expression in EBV (+) and EBV (−) GC (right two panels). Expression of *CD274* mRNA correlated positively with IFN-γ signature expression in EBV (+) GC (P < 0.0001).

Because DNA hypermethylation is a unique epigenetic feature of EBV (+) GC, we first analyzed the DNA methylation status of genes comprising the IFN-γ signature; to do this, we used microarray dataset GSE31789, in which CpG site methylation data for EBV (+) GC (n = 11) and EBV (−) GC (n = 43) are available^5^. In general, IFN-γ signature genes were not hypermethylated in EBV (+) GC (Fig. 2a). Consistent with this, de novo DNA methylation in EBV-infected GC cells (GSE31789) did not induce hypermethylation of IFN-γ signature genes (Fig. 2b)^5^. Importantly, expression of the IFN-γ signature was upregulated in EBV-infected GC cells compared to control cells, suggesting that EBV infection possibly stimulate IFN-γ production (Fig. 2c).

These findings were further supported by gene expression data from microarray dataset GSE51575, which includes EBV (+) GC (n = 12) and EBV (−) GC (n = 14), and the TCGA study, which includes EBV (+) GC (n = 25) and EBV (−) GC (n = 249)^1,20^. The IFN-γ signature was highly expressed in EBV (+) GC compared with EBV (−) GC, with a significant positive correlation between the IFN-γ signature and *CD274* mRNA expression in data from the GSE51575 (Fig. 2d) and TCGA databases (Fig. 2e). These observations are in line with our previous finding that IFN-γ upregulates PD-L1 expression and, importantly, that this mechanism plays a significant role in PD-L1 overexpression, particularly in EBV (+) GC^10^.

### IFN-γ is upregulated by activation of IRF3 in EBV (+) GC

Next, we addressed the mechanism underlying regulation of IFN-γ production in EBV (+) GC. EBV-encoded RNAs (EBERs) or miRNAs induce type I IFNs in EBV-infected cells via activation of IRF3, IRF7, or NF-κB^12^. Here, we focused on IRF3 because it is a key transcription factor that drives antiviral innate immune responses via production of IFN-γ^16^.

**Figure 3:**
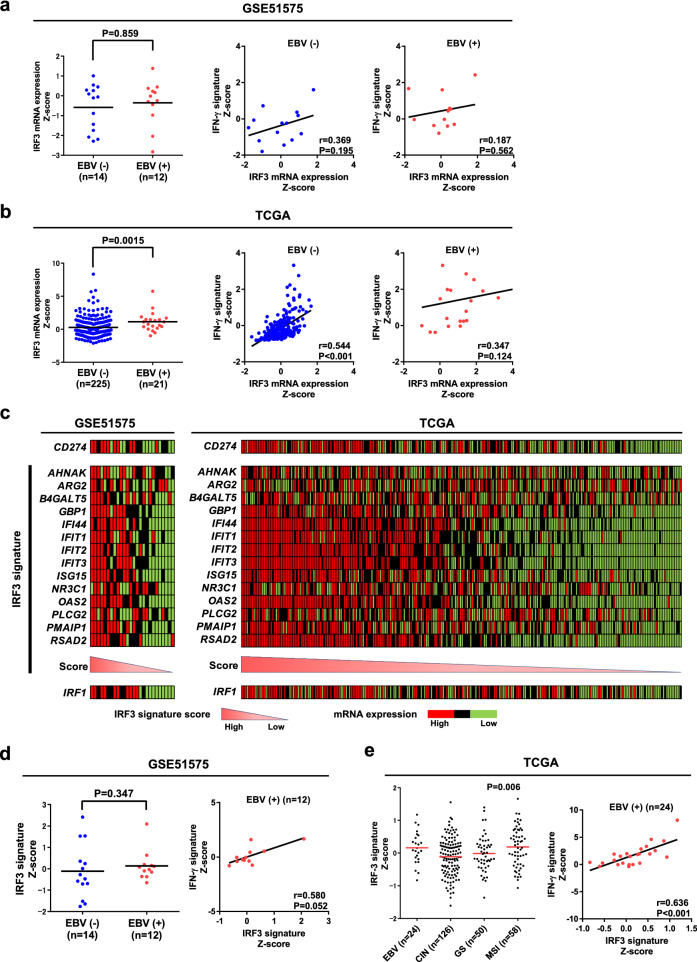
Comparison of IRF3 signature gene expression, *CD274* mRNA expression, and IFN-γ signature gene expression. **(a)** Differential expression of *IRF3* mRNA in EBV (−) and EBV (+) GC (left, GSE51575). *IRF3* mRNA expression was not upregulated in EBV (+) GC compared with EBV (−) GC (P = 0.859). Comparison of IFN-γ signature and *IRF3* mRNA expression in EBV (+) GC (right two panels). The IFN-γ signature was not correlated with *IRF3* mRNA expression in both EBV (+) and EBV (−) GC. **(b)** Differential expression of *IRF3* mRNA in EBV (−) and EBV (+) GC (left, TCGA). Expression of *IRF3* mRNA was upregulated in EBV (+) GC compared with EBV (−) GC (P = 0.0015). Comparison of IFN-γ signature and *IRF3* mRNA expression in EBV (+) GC (right two panels). The IFN-γ signature correlated positively with *IRF3* mRNA expression in EBV (−) GC, but this was not significant in EBV (+) GC (P = 0.124). **(c)** Heatmap depicting the IRF3 signature score and expression of mRNA expression encoding *CD274*, IRF3 signature genes, and *IRF1* (left, GSE51575; right, TCGA). **(d)** Differential expression of IRF3 signature genes between EBV (−) and EBV (+) GC (left), and comparison of IFN-γ signature and IRF3 signature gene expression in EBV (+) GC (right, GSE51575). IRF3 mRNA expression was upregulated in EBV (+) GC compared with EBV (−) GC (P = 0.347). The IFN-γ signature correlated positively (albeit marginally) with IRF3 signature expression in EBV (+) GC (P = 0.052). **(e)** Differential expression of IRF3 signature genes among EBV (+), CIN, GS, and MSI GC (left) cases, and comparison of the IFN-γ signature and IRF3 signature in EBV (+) GC (right, TCGA). The IRF3 signature was upregulated in EBV GC and MSI GC compared with CIN or GS (P = 0.006). The IFN-γ signature correlated positively with the IRF3 signature in EBV (+) GC (P = 0.0008).

Based on previous studies and our own results, we hypothesized that IRF3 is activated in EBV (+) GC, leading to induction of PD-L1/*CD274* expression via IFN-γ. To evaluate this hypothesis, and after we confirmed that IRF3 was not hypermethylated in EBV (+) GC (Supplementary Fig. S4), we investigated expression of IRF3 using data from the GSE51575 dataset and the TCGA study. Expression of IRF3 mRNA was higher in EBV (+) GC than in EBV (−) GC in the TCGA cohort, and expression of IRF3 correlated positively with the IFN-γ signature; however, the trend was not statistically significant (Fig. 3a and b). Therefore, we used an IRF3-upregulated gene signature as a read-out to measure IRF3 activity in data from GSE51575 and the TCGA study. After confirming that 14 genes within the IRF3 signature were generally not hypermethylated in EBV (+) GC (Supplementary Fig. S4), we investigated the correlation between the IRF3 signature and *CD274* mRNA expression in both datasets. Consistent with a previous report, we found that the IRF3 signature correlated positively with *CD274* mRNA expression in GC (Fig. 3c)^21^. The expression of IRF1, which is activated by IFN-γ resulting PD-L1 overexpression, also correlated positively with *CD274* mRNA expression in GC further supported our analysis of public databases (Fig. 3c)^22^. Expression of IRF3 signature genes was higher in EBV (+) GC than in EBV (−) GC, and correlated positively (albeit marginally) with the IFN-γ signature in EBV (+) GC; however, the results did not reach statistical significance, probably due to the small number of samples in the GSE51575 cohort (Fig. 3d). Analysis of the TCGA study revealed higher expression of the IRF3 signature in EBV (+) GC than in CIN or gnomically stable (GS) cases, and a significant positive correlation with the IFN-γ signature in EBV (+) GC (Fig. 3e). These results suggest that overexpression of PD-L1 in EBV (+) GC is induced by high IRF3 activity via IFN-γ.

### IRF3 is activated in EBV (+) GC

**Figure 4:**
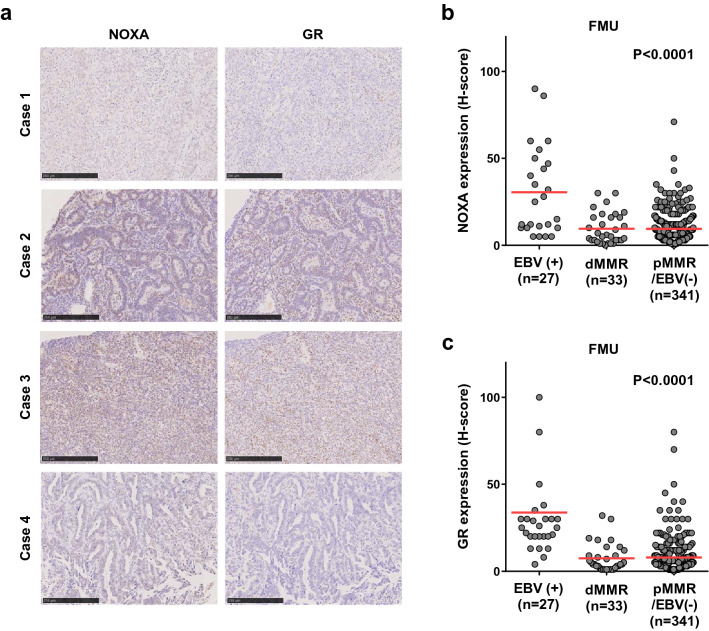
Differential expression of IRF3 signature genes among EBV (+), dMMR, and pMMR/EBV (−) gastric cancer (GC) samples from FMU cohort. **(a)** Representative images showing IHC staining for NOXA in Case 1 (H-score = 10), 2 (H-score = 60), 3 (H-score = 35), and 4 (H-score = 1), and GR in Case 1 (H-score = 25), 2 (H-score = 160), 3 (H-score = 70), and 4 (H-score = 6). Case number is showing the same case as in Fig. 1a,b. Scale bars = 250 μm. (b) NOXA was expressed more strongly in EBV (+) than in dMMR or pMMR/EBV (−) GC in the FMU cohort (P < 0.0001). **(c)** GR was expressed more strongly in EBV (+) than in dMMR or pMMR/EBV (−) GC in the FMU cohort (P < 0.0001).

Finally, to confirm the above findings in clinical samples, we performed IHC staining of representative IRF3 signature genes in samples of FMU GC. NOXA [encoded by phorbol-12-myristate-13-acetate-induced protein 1 (*PMAIP1*)] was expressed more strongly in EBV (+) GC than in dMMR or pMMR/EBV (−) GC in the FMU cohort (Fig. 4a,b and Supplementary Fig. S5). In addition, glucocorticoid receptor (GR) [encoded by nuclear receptor subfamily 3 group C member 1 (*NR3C1*)] was also expressed more strongly in EBV(+) GC than in dMMR or pMMR/EBV (−) GC in the FMU cohort (Fig. 4a,c and Supplementary Fig. S5). These results strongly suggest that the IRF3 signature is activated in EBV (+) GC.

## Discussion

Here, we demonstrated that PD-L1 overexpression in EBV (+) GC is caused mainly by two different mechanisms: *CD274* focal amplification and IFN-γ-mediated signaling, including via IRF3 activation. The mechanisms regulating PD-L1 expression in GC cells have been described in several studies; additionally, we recently reported that PD-L1 expression is associated with CD8 T cell infiltration and IFN-γ production within the tumor microenvironment of GC^9,10^. Although a recent study reported that the Epstein-Barr virus nuclear antigen 2 affects PD-L1 expression in diffuse large B-cell lymphoma, the specific mechanism underlying PD-L1 expression in EBV (+) GC remains unclear^23^. Here, we reveal that high expression of PD-L1 by tumor cells in EBV (+) GC is due to high levels of *CD274* focal amplification in the absence of CD8 lymphocyte infiltration. By contrast, relatively high expression of PD-L1 in tumor and infiltrating immune cells correlated with IFN-γ production via IRF3 activation.

Amplification of *CD274*, leading to overexpression of PD-L1 in tumor cells, is an interesting characteristic of GC, particularly EBV (+) GC^24,25^. The data presented herein, along with TCGA analysis, reveal that focal and high level amplification of *CD274* results in high expression of PD-L1/*CD274* in EBV (+) GC tumors. This result is in line with our previous study showing that a small subset of SCLC harbors focal and high level *CD274* amplification, resulting in high expression of PD-L1^8^. Our cohort and TCGA data revealed that EBV (+) GC tumors with high *CD274* amplification and high PD-L1 expression were not infiltrated by CD8 + lymphocytes. These results indicate that constitutive oncogenic signaling due to chromosomal alterations and amplifications is independent of inflammatory signals in the tumor microenvironment of EBV (+) GC. Different from other GC subtypes, EBV (+) GC is associated with EBV infection; therefore, innate antiviral immune responses in EBV (+) GC contribute to PD-L1 overexpression. By contrast, EBV (+) GC with non-focal, arm-level copy number gain of 9p did not exhibit high *CD274* mRNA expression when compared with that in tumors with focal amplification, presumably resulting in relatively high PD-L1 expression^8^. Of note, although arm-level 9p gain occurs in EBV (+) GC, this was not the main structural aberration in chromosome 9p in GC; arm-level 9p loss is more common, resulting in loss of *CDKN2A*^26^.

In contrast to constitutive PD-L1 expression by tumor cells due to *CD274* amplification, PD-L1 was induced in tumors and infiltrating immune cells in EBV (+) GC in response to adaptive immune resistance. Through this response, various cytokines (such as interleukins, TNF, and IFNs) are produced or released from infected cells to induce or maintain PD-L1 expression^12^. Among these cytokines, IFN-γ is the most potent inducer of PD-L1 expression in GC tumors^9,10^. Based on these findings, we hypothesized that IFN-γ-mediated signals are induced by IRF3, which could generate cytokines that facilitate viral infection and IFNs that inhibit viral infections. In EBV (+) GC, IRF3 is activated by phosphorylation (to yield pIRF3) through the TLR or RIG-I signaling pathways, which are triggered by EBER1, EBER2, EBV-encoded miRNAs, or other non-coding RNAs^12,13^. The fact that ISH for EBER1 is used commonly as a diagnostic test for EBV infection suggests that EBER1 is expressed at levels sufficient to stimulate the TLR or RIG-I pathways in EBV-infected cells, but expression status of others which could stimulate IRF3 expression were unknown in EBV (+) GC.

The present study focused on IRF3 activity, as measured by transcription and translational production of its target genes, and confirmed that it correlated with *CD274* expression^21^. In EBV-infected tumors, IRF3 acts as key downstream transcriptional effector of inflammation and immunity thorough TLR3 and TLR4 signaling in macrophages. Using two independent cohorts, we demonstrated that the IRF3 signature tended to be higher in EBV (+) GC than in other types of GC, and that it correlated positively with the IFN-γ signature and with *CD274* mRNA expression^21^. In addition, we further confirmed these findings in clinical samples by showing that NOXA/*PMAIP1* and GR/*NR3C1*, which are components of the IRF3 signature gene set, were expressed at higher levels in EBV (+) GC than in EBV (−) GC. Of note, the IRF3 signature was also upregulated in MSI cases in TCGA (Fig. 3e) and also correlated positively with the IFN-γ signature (Supplementary Fig. S6a). However, since genes within the IRF3 signature in MSI GC are frequently mutated, accumulation of gene mutations may contribute to activation of the IRF3 signature in MSI GC (Supplementary Fig. S6b). To support this consideration, it was confirmed that NOXA encoded by *PMAIP1* and GC encoded by *NR3C1* in which IRF3 signature genes were not strongly expressed in dMMR than EBV (+) GC (Fig. 4b and c).

Based on the results of the ATTRACTION-2 study, immune checkpoint blockers targeting the PD-1 axis have been approved for treatment of GC in many countries, including Japan, South Korea, Singapore, and Switzerland^6,27^. Indeed, metastatic GC patients with MSI-high, high PD-L1 CPS, and EBV (+) tumors show a favorable response to anti-PD-1 mAbs used as second- or higher-line treatments^7^. As for GC, patients with other cancers showing high PD-L1 expression and a high mutational load tend to have a favorable clinical course^28,29^. Importantly, since there is a general positive correlation between EBV (+) GC and PD-L1 expression, and these patients show the best response to anti-PD-1 mAbs. However, in this study, we showed the possibility that PD-L1 overexpression associated with the presence high level *CD274* focal amplification without lymphocyte infiltration would not show a favorable response to anti-PD-1 mAbs^30^. Additionally, our results recommend an EBV test to detect EBV infection for all patients with GC to prevent loss of treatment opportunity^7^. Of importance, because some EBV (+) GC cases with conventional-type adenocarcinoma exhibit well to moderately differentiated adenocarcinoma without lymphocyte infiltration and lase pattern, it is hard to detect EBV infection histologically (Supplementary Fig. S1).

In conclusion, we have identified the mechanism underlying overexpression of PD-L1 in EBV (+) GC. We show that high expression of PD-L1 in tumor cells is due to high level *CD274* focal amplification without lymphocyte infiltration. On the contrary, relatively high level expression of PD-L1 in tumor and infiltrating immune cells is due to IFN-γ-mediated signaling via activation of IRF3.

## Materials and methods

### Clinical samples of patients

The study included 401 surgical specimens collected from GC patients who underwent surgical resection at Fukushima Medical University Hospital (FMU cohort) between 2002 and 2018. This cohort included the test cohort in our previous study consisted 200 patients^19^. Samples were subjected to EBER in situ hybridization (ISH) to assess EBV infection status and immunohistochemical (IHC) staining for mismatch repair (MMR) proteins, MLH1, MSH2, MSH6, and PMS2^19^. Data on age, sex, TNM stage (8th classification), and pathological diagnosis, including microscopic feature of EBV (+) GC such as lymphoepithelioma-like carcinoma (LELC), carcinoma with Crohn’s disease-like lymphoid, and conventional type adenocarcinoma, were retrospectively collected. The carcinomas at the time of primary tumor resection were staged according to the Union for International Cancer Control classification. The study was approved by the ethics committee of Fukushima Medical University. All patients provided written informed consent. All experiments were carried out in accordance with the approved study plan and relevant guidelines.

### Microarray data analysis

All microarray data are publicly available from the Gene Expression Omnibus (GEO) database (http://www.ncbi.nlm.nih.gov/geo). We utilized microarray gene methylation profiles from 54 patients with GC and EBV-infected GC cells, using Akata system of recombinant EBV, deposited as GSE31789 on the basis of Illumina Infinium HumanMethylation27 BeadChip^5^ and analyzed according to our previous study^31^. We also utilized mRNA expression profiles from EBV-infected cells, deposited as GSE31787 on the basis of Affymetrix GeneChip Human Genome U133 Plus 2.0 oligonucleotide array^5^. We also utilized the expression profiles from 26 patients with GC deposited as GSE51575 on the basis of Agilent-028004 SurePrint G3 Human GE 8 × 60K Microarray^20^. The normalized expression values were obtained from each dataset and were not processed further. If a gene is represented by multiple probe sets, the methylation and expression values of multiple probes were averaged.

### TCGA database analysis

Copy number alteration, gene mutation, or expression data of GC patients were obtained from TCGA’s cBioPortal database (http://www.cbioportal.org/). Those data of *CD274,* IFN-γ and IRF3 signature genes in GC patients were used for the analyses. For the expression data, RNA-sequencing data that was normalized by RSEM method was used for the analyses. For the clinical tumors, a multi-omics study of 295 GC patients including 269 EBV (−) and 26 EBV (+) GC was selected for the analyses^1^.

### Immunohistochemical staining and evaluation

IHC staining of formalin-fixed, paraffin-embedded (FFPE) histological sections (4 μm thick) was performed using a polymer peroxidase method, as previously performed^19,32^. Briefly, after deparaffinization and rehydration, the sections were treated with 0.3% hydrogen peroxide in methanol for 30 min to block endogenous peroxidase activity. After rinsing in PBS, the sections were incubated with anti-PD-L1 antibody (#13684; E1L3N; 1:200 dilution; Cell Signaling Technology, Danvers, MA, USA), anti-CD8 antibody (1:1000 dilution; Cell signaling Technology), anti-NOXA (1:1000 dilution; Novus, Centennial, CO, USA) and anti-GR (1:400 dilution; Cell Signaling Technology) at 4 °C overnight. An additional wash in PBS was followed by treatment with a peroxidase-labeled polymer conjugated to goat anti-rabbit immunoglobulins (ENvision + kit; Dako, Agilent, Santa Clara, CA, USA) as the secondary antibody for 30 min at room temperature. The staining was visualized with diaminobenzidine, followed by counterstaining with hematoxylin. PD-L1 expression was determined by using combined positive score (CPS), which is the number of PD-L1 staining cells among tumor cells, lymphocytes, and macrophages divided by the total number of viable tumor cells. Evaluation of NOXA and GR expressions were defined as positive or negative expression and performed as previously described^19^. IHC staining for MMR protein and integration of EBV by EBER-ISH were performed, as previously described^19^.

### Statistical analysis

Mann–Whitney U test were used to determine differences between two variables. Spearman’s correlation was used to evaluate the correlations between levels of expression. Pearson correlation analysis was used to examine the relationships between two parameters. ANOVA test was used for multiple comparisons. All statistical analyses were conducted using GraphPad Prism v7.0 (Graphpad Software Inc., San Diego, CA, USA) or JMP 10 software (SAS Institute, Cary, NC, USA). All P values were two-sided, and P values less than 0.05 were considered statistically significant.

## Supplementary Information

Supplementary Figures.
