# Supplementary material for: PD-L1 overexpression in EBV-positive gastric cancer is caused by unique genomic or epigenomic mechanisms
Source: Sci Rep. 2021 Jan 21;11:1982. doi: 10.1038/s41598-021-81667-w (PMC7820576; doi:10.1038/s41598-021-81667-w)
Supplement: Supplementary file 1 — Supplementary Figures. [file 41598_2021_81667_MOESM1_ESM.pdf]

PD-L1 overexpression in EBV-positive gastric cancer is caused by unique genomic or epigenomic mechanisms

Hiroshi Nakano[1], Motonobu Saito[1*], Shotaro Nakajima[1,2], Katsuharu Saito[1], Yuko Nakayama[3], Koji Kase[1], Leo Yamada[1], Yasuyuki Kanke[1], Hiroyuki Hanayama[1], Hisashi Onozawa[1], Hirokazu Okayama[1], Shotaro Fujita[1], Wataru Sakamoto[1], Zenichiro Saze[1], Tomoyuki Momma[1], Kosaku Mimura[1,4], Shinji Ohki[1], Akiteru Goto[5], Koji Kono[1]

[1]Department of Gastrointestinal Tract Surgery, Fukushima Medical University School of Medicine, Fukushima 960-1295, Japan
[2]Department of Medical Electrophysiology, Fukushima Medical University School of Medicine, Fukushima 960-1295, Japan
[3]First Department of Surgery, Faculty of Medicine, University of Yamanashi, Yamanashi 409-3898, Japan
[4]Department of Blood Transfusion and Transplantation Immunology, Fukushima Medical University School of Medicine, Fukushima 960-1295, Japan
[5]Department of Cellular and Organ Pathology, Graduate School of Medicine, Akita University, Akita 010-8543, Japan

***Correspondence:** Motonobu Saito
Department of Gastrointestinal Tract Surgery, Fukushima Medical University School of Medicine
1 Hikarigaoka, Fukushima City, 960-1295, Japan
TEL: +81-24-547-1259; FAX: +81-24-547-1980; E-mail: moto@fmu.ac.jp

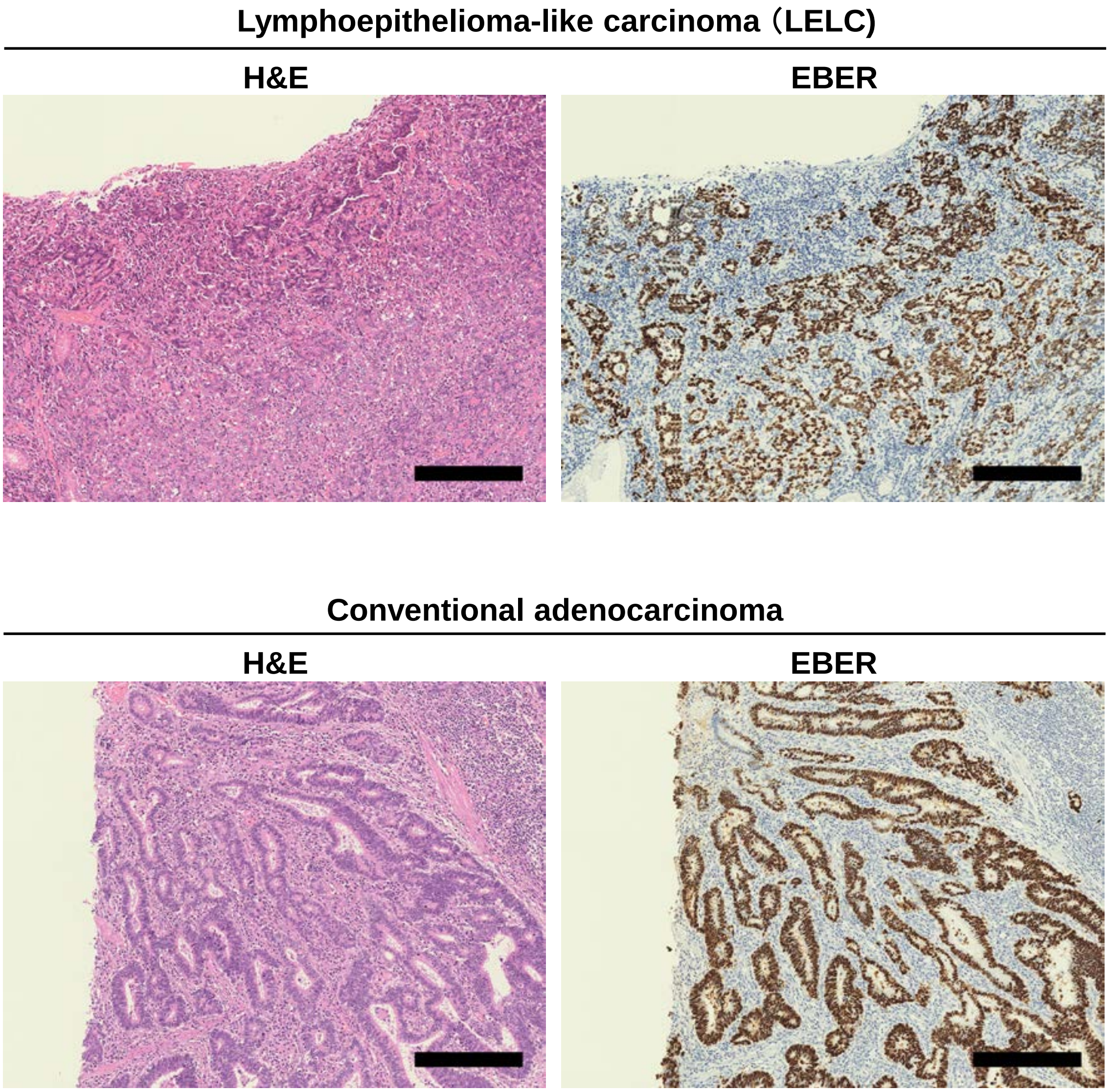

Supplementary Fig. S1. Representative images showing IHC staining for H&E and EBER-ISH. H&E, Hematoxylin and Eosin; EBER-ISH, EBV-encoded small RNAs-in situ hybridization. Scale bars=250μm

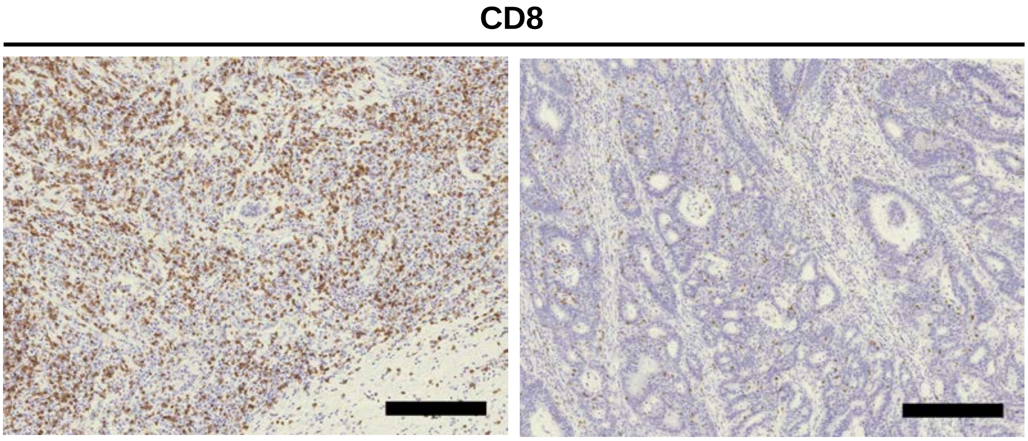

Supplementary Fig. S2. Representative images showing IHC staining for CD 8 T cells in EBV (+) GC cases from the FMU cohort. Cases with a high (25%, left) and low (0%, right) percentage of infiltrating CD8+ lymphocytes (0%, right) are shown. Scale bars = 250 μm.

Supplementary Fig. S3

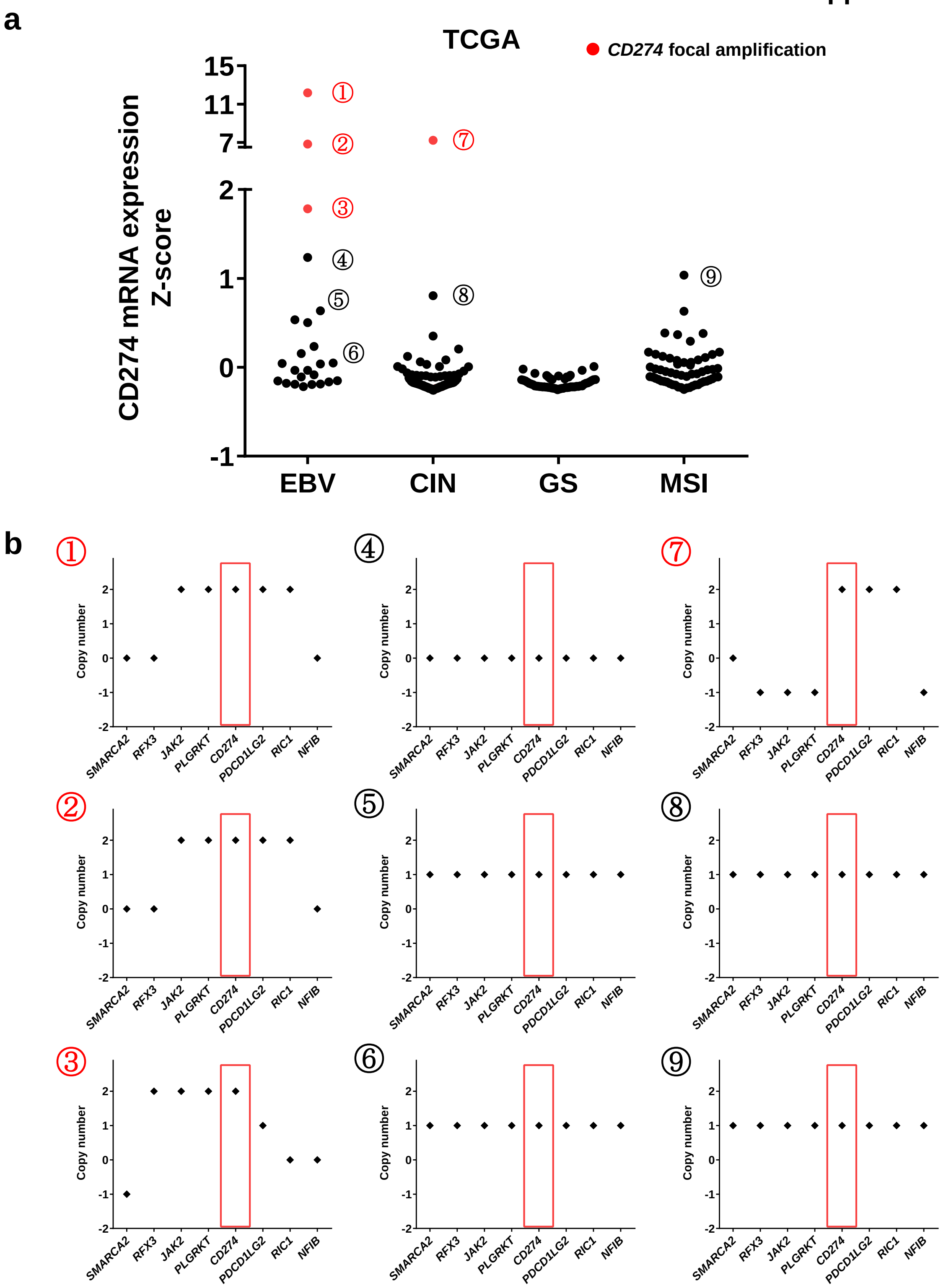

Supplementary Fig. S3. (**a**) *CD274* mRNA expression among EBV (+), , CIN, GS, and MSI GC (TCGA). Red point showing tumor with *CD274* focal amplification. (**b**) The copy number states of representative cancer-related genes that mapped telomeric and centromeric to *CD274* on 9p24.1. Cases 1, 2, 3, and 7 showed high *CD274* mRNA expression by focal and high-level amplification of the segment containing *CD274*. Cases 5, 8, and 9 showed relatively high *CD274* expression with non-focal and arm-level copy number gain of 9p.

a

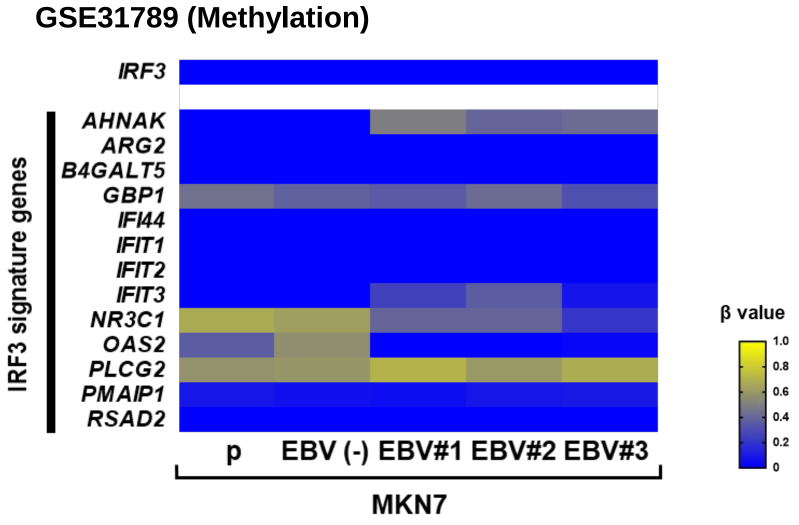

b

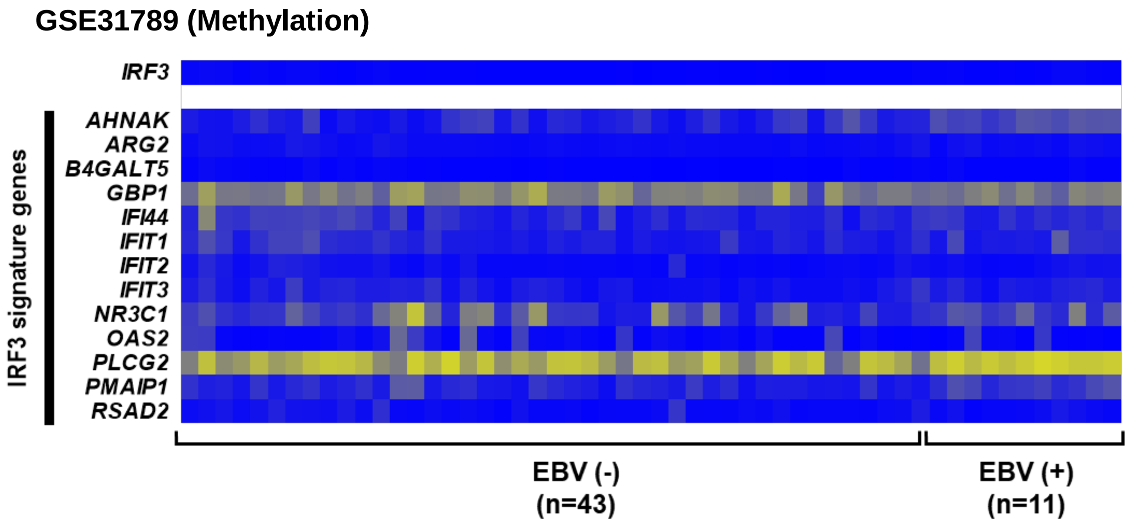

Supplementary Fig. S4. (**a**) DNA methylation status of IRF3 and IRF3 signature genes (13 genes) among the parent (MKN7 p), mock [MKN7 EBV (-)], and EBV-infected clones (MKN7 EBV#1, EBV#2, and EBV#3) in MKN7 cells (GSE31789). DNA hypermethylation was not observed in IRF3 signature genes. The β value, 0.00 to 1.00, reflects the methylation level of the individual CpG site. (**b**) DNA methylation status of IRF and IRF3 signature genes (13 genes) between EBV (-) (n = 43) and EBV (+) (n = 11) GC (GSE31789). DNA hypermethylation was not observed in IRF3 signature genes. The β value, 0.00 to 1.00, reflects the methylation level of the individual CpG site.

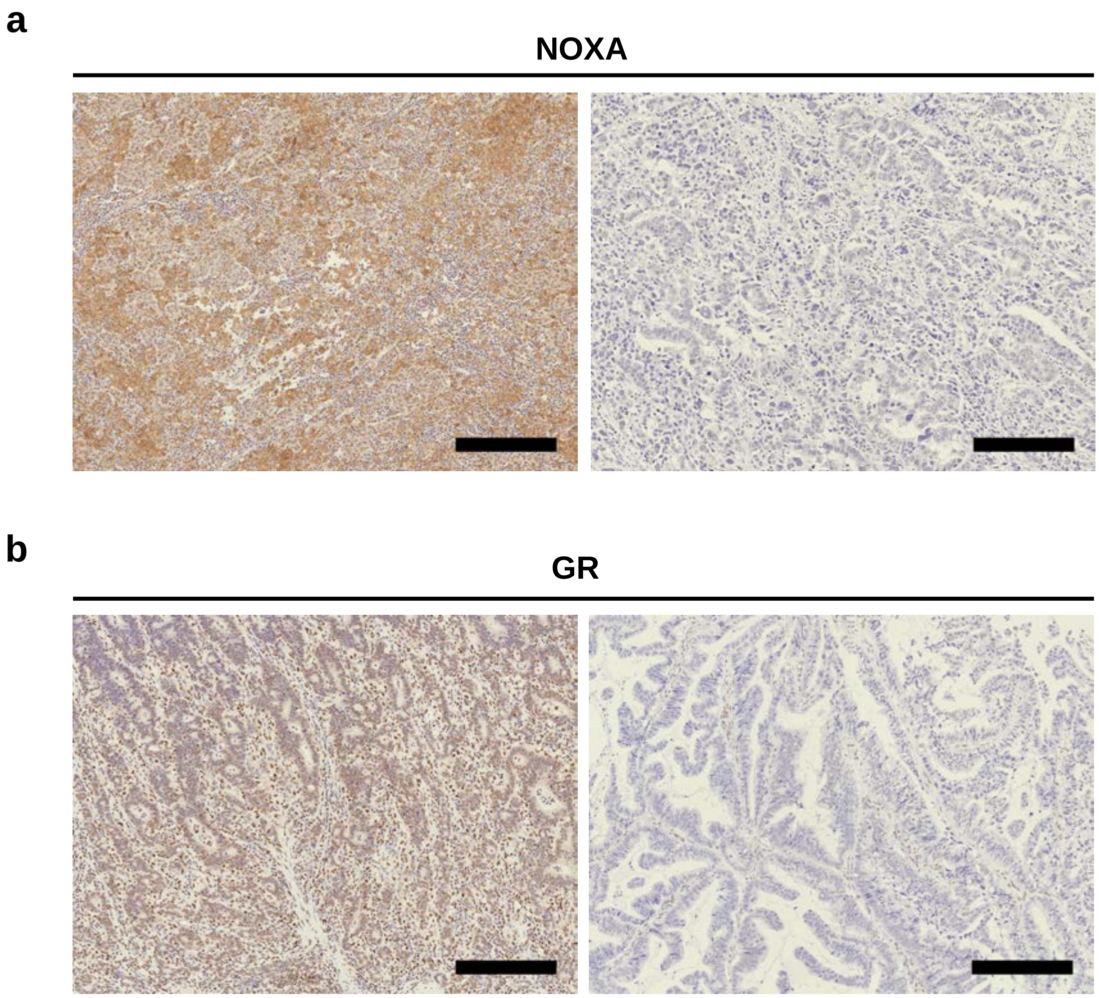

Supplementary Fig. S5. Representative images showing IHC stainng for NOXA and GC. (**a**) IHC staining for NOXA high (left) or low (right) expressions in GC. (**b**) Representative images showing IHC staining for GR high (left) or low (right) expressions in GC. Scale bars = 250 μm.

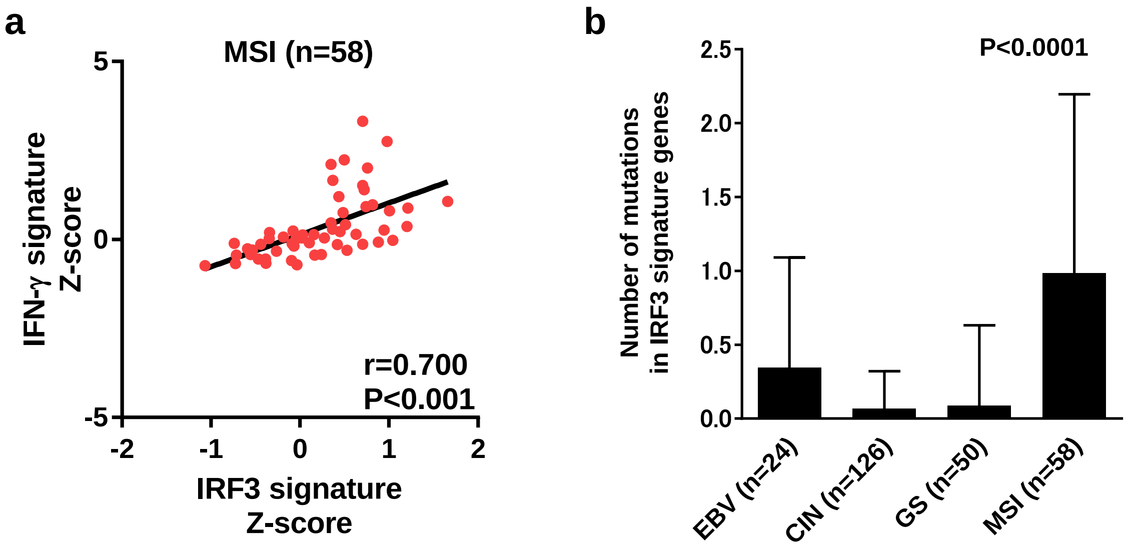

Supplementary Fig. S6. Molecular features of IRF3 signature in MSI GC. (**a**) Comparison between IFN-γ signature and IRF3 signature in MSI GC (TCGA). IFN-γ signature was positively significantly correlated with IRF3 signature in MSI GC (P < 0.001). (**b**) Average number of gene IRF3 signature gene mutations among EBV (+), CIN, GS, and MSI GC (TCGA). Gene mutations was highly accumulated in MSI GC (P < 0.001).
